# Glucocorticoids Inhibit Basal and Hormone-Induced Serotonin Synthesis in Pancreatic Beta Cells

**DOI:** 10.1371/journal.pone.0149343

**Published:** 2016-02-22

**Authors:** Moina Hasni Ebou, Amrit Singh-Estivalet, Jean-Marie Launay, Jacques Callebert, François Tronche, Pascal Ferré, Jean-François Gautier, Ghislaine Guillemain, Bernadette Bréant, Bertrand Blondeau, Jean-Pierre Riveline

**Affiliations:** 1 INSERM, UMR_S 1138, Centre de Recherche des Cordeliers, F-75006, Paris, France; 2 Sorbonne Universités, UPMC, Univ Paris 06, UMR_S 1138, Centre de Recherche des Cordeliers, F-75006, Paris, France; 3 Université Paris Descartes, Sorbonne Paris Cité, UMR_S 1138, Centre de Recherche des Cordeliers, F-75006, Paris, France; 4 INSERM U942, Assistance Publique–Hôpitaux de Paris (AP-HP), Hôpital Lariboisière, Service de Biochimie, Paris, France; 5 CNRS UMR INSERM 952-CNRS 7224, Paris, France; 6 Department of Diabetes and Endocrinology, Hôpital Lariboisière, AP-HP, Paris, France; 7 Université Paris Diderot, Paris, France; CRCHUM-Montreal Diabetes Research Center, CANADA

## Abstract

Diabetes is a major complication of chronic Glucocorticoids (GCs) treatment. GCs induce insulin resistance and also inhibit insulin secretion from pancreatic beta cells. Yet, a full understanding of this negative regulation remains to be deciphered. In the present study, we investigated whether GCs could inhibit serotonin synthesis in beta cell since this neurotransmitter has been shown to be involved in the regulation of insulin secretion. To this aim, serotonin synthesis was evaluated in vitro after treatment with GCs of either islets from CD1 mice or MIN6 cells, a beta-cell line. We also explored the effect of GCs on the stimulation of serotonin synthesis by several hormones such as prolactin and GLP 1. We finally studied this regulation in islet in two in vivo models: mice treated with GCs and with liraglutide, a GLP1 analog, and mice deleted for the glucocorticoid receptor in the pancreas. We showed in isolated islets and MIN6 cells that GCs decreased expression and activity of the two key enzymes of serotonin synthesis, Tryptophan Hydroxylase 1 (Tph1) and 2 (Tph2), leading to reduced serotonin contents. GCs also blocked the induction of serotonin synthesis by prolactin or by a previously unknown serotonin activator, the GLP-1 analog exendin-4. In vivo, activation of the Glucagon-like-Peptide-1 receptor with liraglutide during 4 weeks increased islet serotonin contents and GCs treatment prevented this increase. Finally, islets from mice deleted for the GR in the pancreas displayed an increased expression of Tph1 and Tph2 and a strong increased serotonin content per islet. In conclusion, our results demonstrate an original inhibition of serotonin synthesis by GCs, both in basal condition and after stimulation by prolactin or activators of the GLP-1 receptor. This regulation may contribute to the deleterious effects of GCs on beta cells.

## Introduction

Diabetes mellitus (DM) is one of the most frequent complications of chronic exposure to glucocorticoid (GCs) especially during a Cushing's syndrome (CS) or after treatment with high doses of GCs. Its prevalence is considered to range from 20 to 50% [1]. In general, the prevalence of glucose metabolism alterations including impaired fasting glycaemia (IFG) and impaired glucose tolerance (IGT) reaches 70% after GCs exposure [2]. More generally, type 2 diabetes (T2D) is associated with a subtle hypercortisolism, suggesting a causal role for GCs in T2D [3]. These abnormalities of glucose metabolism occur as a consequence of insulin resistance and impaired insulin secretion induced by GCs excess [4]. These alterations have been studied in vitro using isolated islets and beta-cell lines. Such studies demonstrated that GCs directly inhibit beta-cell function [5] and reduce beta cell mass by inducing apoptosis [6]. However, the molecular mechanisms of these effects remain unclear. Therefore, unraveling the mechanisms by which GCs alter glucose homeostasis but more specifically insulin secretion could lead to a better understanding of the beta-cell alterations after GCs excess and more generally in type 2 diabetes.

GCs are steroid hormones produced by the zona fasciculata of adrenals under the control of the hypothalamic-pituitary-adrenal axis, secreted according to a circadian rhythm and in adaptive situations such as stress or fasting which lead to energy store mobilization [4]. They act on their target tissues through the binding to the glucocorticoid receptor, GR, which is expressed in almost every cell. In the absence of the hormone, GR is restrained in the cytoplasm; upon binding of its ligand, GR migrates to the nucleus where it acts as a transcription factor and activates or inhibits the expression of target genes [7]. Thus, the comprehensive understanding of how beta cells are controlled by GCs and how this control impacts on the regulation of glucose homeostasis requires the identification of GCs targets in these cells. Previous studies have shown that GCs decrease the expression of the glucose transporter [8, 9] and glucokinase [10] in pancreatic beta cells. Others have observed deleterious effects of GCs on membrane depolarization [11] or exocytosis of insulin-containing vesicles [12, 13].

Another hypothesis is that GCs may inhibit pathways that are crucial for beta-cell function. Among these pathways, serotonin and its synthesis in beta cells have been recently described as important modulators of insulin secretion and beta-cell mass [14, 15]. Serotonin (5-hydroxytryptamine, 5-HT) is derived from the amino acid tryptophan. In serotonin producing cells, tryptophan is hydroxylated by the rate limiting enzyme tryptophan hydroxylase (Tph) and subsequently decarboxylated by aromatic acid decarboxylase [16]. There are two main pools of serotonin: one pool is synthesized in the brainstem and one in peripheral tissues. In both locations, serotonin synthesis relies on the enzyme tryptophan hydroxylase, which is encoded by two different genes, Tryptophan hydroxylase 1 (Tph1) and Tryptophan hydroxylase 2 (Tph2) expressed in peripheral tissues and in the brain, respectively. In the brain, serotonin serves as a neurotransmitter where it regulates multiple physiological aspects, including behavior, learning, mood and appetite. However, the brain-derived serotonin accounts only for around 5% of total body serotonin. The remaining 95% of serotonin are produced in the peripheral organs, stored in the platelets and released to regulate several major functions. Serotonin is also synthesized by pancreatic beta cells and is thought be secreted together with insulin [17]. Its role in beta cells was elusive but two recent studies described serotonin as a major regulator of both beta-cell mass and function. The first study showed that intracellular serotonin participates to the regulation of insulin exocytosis while extracellular serotonin activates receptors that also modulate insulin secretion [15]. The other reported that increase of serotonin synthesis and activation of specific receptor promote beta-cell proliferation observed during pregnancy in mice [14]. Interestingly, GCs inhibit serotonin synthesis in the raphe nuclei, and conversely, the knockdown of GR in the zebra fish was associated with up-regulated Tph 2 expression [18]. Therefore, we asked whether GCs could also inhibit serotonin synthesis in beta cells.

We evaluated the effect of GCs on Tph1 and 2 expression and serotonin synthesis in beta cell lines and isolated islets. We demonstrated here that GCs inhibit Tph1 and 2 expression, decrease Tph activity and lead to reduced serotonin contents. We therefore provide evidences that serotonin synthesis in beta cells is under the control of GCs.

## Research Design and Methods

### Cell culture

MIN6 cells were cultured as previously described [19] in 25 mM glucose DMEM supplemented with 15% fetal bovine serum, 1% penicillin and streptomycin. Isolated islets were cultured in 11 mM glucose RPMI supplemented with 10% fetal bovine serum, 1% penicillin and streptomycin. Islets and MIN6 cells were treated with 10^−7^M Dexamethasone (Dex, Sigma-Aldrich, MO, USA), with or without 1 μg/ml human prolactin (PRL, gift of Latif Rachdi, Inserm, Paris) or 100 ng/ml exendin-4 (Sigma).

### Mouse husbandry

Animals were bred and raised under standard animal housing conditions, in a 12-h light/dark cycle (7am–7pm), temperature (22±1°C) and humidity (60±5%). Food (standard chow) and water were available *ad libitum*. All experiments were performed in accordance with French guidelines for care of laboratory animals. The experiments were examined and approved by the Regional Ethics Committee in Animal Experiment N°3 of Ile-de-France region (reference p3/2008/012). Mice that lack the GR in beta cells (GR^Lox/Lox^—PdxCre mice) have been previously described [20]. Since it has been recently shown that the presence of the PdxCre transgene is sufficient to induce a beta-cell phenotype [21], we chose to analyze mice that carry the floxed allele only (GR^Lox/Lox^), mice that carry the PdxCre transgene but wild type alleles of the GR (GR^+/+^—PdxCre) and mice that possess floxed alleles and the PdxCre transgene (GR^Lox/Lox^–PdxCre). All mice were backcrossed on a C57BL/6J genetic background for 12 generations. CD1 mice were provided by Charles River (Charles River France). Mice were anesthetized using 1% ketamine and 0.2% xylazine. Mice were killed by cervical dislocation. Only male mice were studied.

### In vivo glucocorticoids and GLP-1 analog treatments

Experiments were performed on 8 week-old C57BL/6J mice from Charles River (Charles River, Saint-Germain Nuelles, France). Animals received corticosterone (100 μg/ml, Sigma France) or vehicle (1% ethanol) in the drinking water. Half of the mice in each group were given once daily intraperitoneal injections of liraglutide, a GLP-1R agonist (Novo Nordisk La Défense, France) and the other half were given saline (0.9% NaCl). Liraglutide was given in increasing doses from 0.15 mg per kg body weight with a daily increment of 0.025 mg/kg until the final dose of 0.3 mg per kg body weight was reached, as previously described [22]. Treatments were performed for 4 weeks.

### Mouse islet isolation

Mouse islets were isolated with collagenase (1mg/ml, Sigma-Aldrich) solution, separated on Histopaque (Sigma-Aldrich) gradient and handpicked under a binocular stereo microscope (Leica Microsystems GmbH, Wetzlar, Germany), as previously described [23].

### RNA extraction, cDNA synthesis and qPCR

Total RNAs were extracted from MIN6 cells or islets using RNeasy Plus extraction kit (Qiagen, Hilden, Germany) according to the manufacturer guidelines. RNAs were reverse transcribed into cDNA using Superscript II reverse transcriptase (Invitrogen). Gene expression was quantified by real-time PCR with SybrGreen supermix in a MyIQ thermocycler (Biorad, CA, USA). Control MIN6 cells and islets cDNAs were diluted and used as a standard curve. Gene expression was normalized to the 18S ribosomal RNA. Primers were designed to span two exons whenever possible. Primer sequences are available upon request.

### Serotonin assay

Cells or islets were washed and homogenized in 200μl ice-cold 0.1 M acetic acid buffer containing 10 mM sodium metabisulfite, 10mM EDTA and 10 mM ascorbic acid. After centrifugation, the supernatant was passed through a 10000 MW filter (Nanosep 1N kDa, Pall). A 20 μl sample aliquot was subsequently analyzed for serotonin using high-performance liquid chromatography as previously described [24].

### Tryptophan hydroxylase (Tph) activity assay

Tph activity was determined by a radioenzymatic assay using [^3^H]-tryptophan as substrate and conducted under conditions validated for neuroendocrine studies [25].

### Western blot

Total proteins were separated on SDS-PAGE gels and transferred to nitrocellulose membranes. Membranes were incubated with an antibody raised in rabbit against Tph1 (Novus Biologicals). Band quantification was performed using a Kodak Imager (Kodak, NY, USA).

### Statistical analysis

All results are expressed as means ± SD. The statistical significance of variations was tested by a Mann-Whitney non-parametric test or by a two way-ANOVA test when multiple groups were compared. P values < 0.05 were considered significant.

## Results

### GCs inhibit Tph1 and Tph2 expression and serotonin synthesis in beta cells

**Fig 1:**
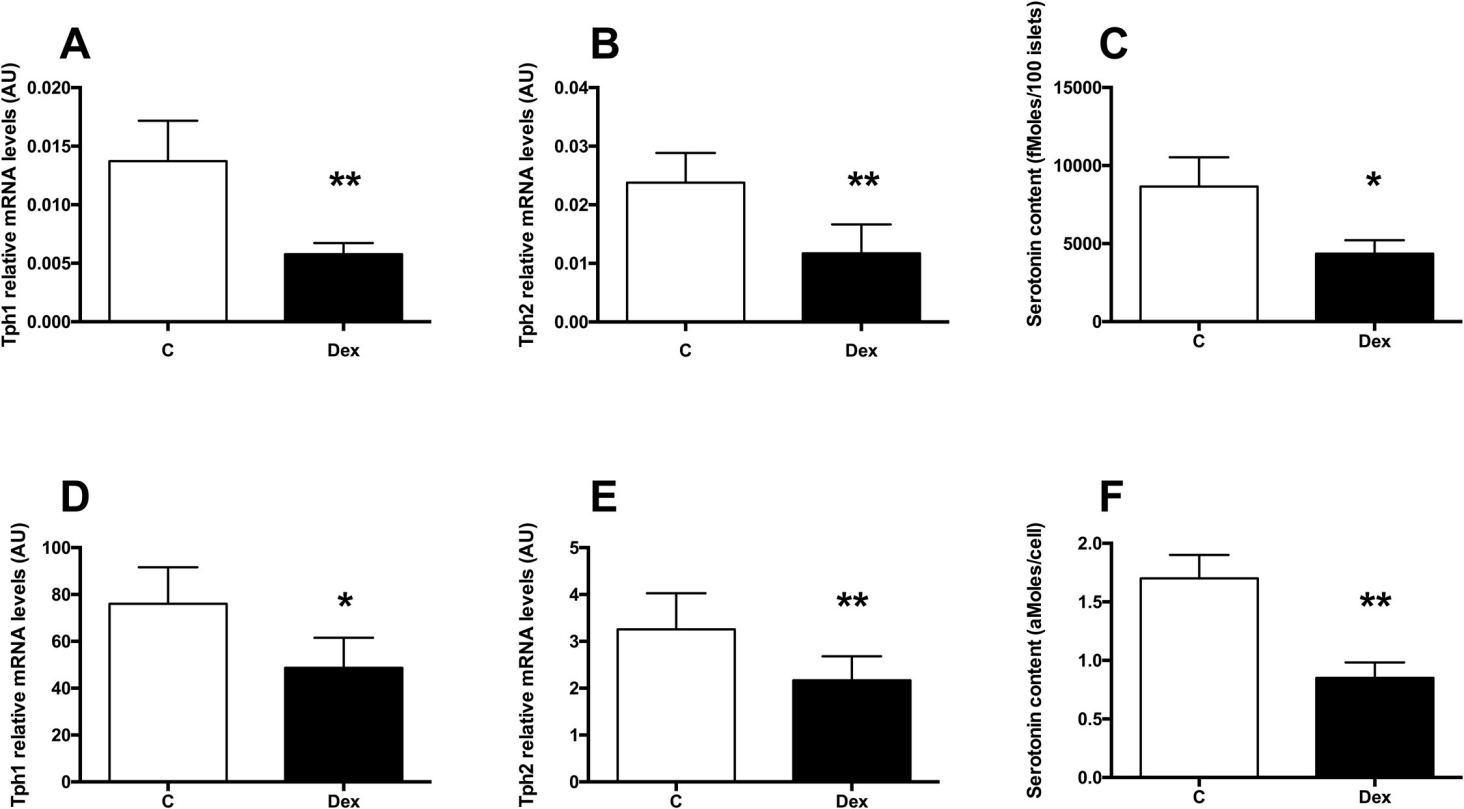
GCs inhibit Tph1 and Tph 2 expression and serotonin synthesis in beta cells. (A) Tph1 and (B) Tph 2 mRNA levels in isolated wild type mouse islets cultured in control conditions (white bars) or treated with 10^−7^M Dexamethasone for 24h (black bars). (C) Serotonin contents in mouse islets cultured in control conditions (white bars) or treated with 10^−7^M Dex for 24h (black bars). (D) Tph1 and (E) Tph 2 mRNA levels in MIN6 cells cultured in control conditions (white bars) or treated with 10^−7^M Dex for 24h (black bars). (F) Serotonin contents in MIN6 cells cultured in control conditions (white bars) or treated with 10^−7^M Dex for 24h (black bars). Results are expressed as means ± SD for n = 4 independent experiments. * p<0.05 and ** p<0.01 when comparing Dex-treated *vs* control islets or MIN6 cells, using a Mann-Whitney non parametric test.

To decipher how GCs control beta cells, we asked whether GCs could inhibit the synthesis of serotonin in beta cells. We tested this hypothesis in isolated islets from CD1 mice and in MIN6 cells. In cultured mouse isolated islets, a 24h Dexamethasone (Dex, a synthetic GCs) treatment decreased Tph1 (Fig 1A) and Tph2 (Fig 1B) mRNA levels leading to decreased serotonin contents (Fig 1C). Similar results were obtained in MIN6 cells (Fig 1D, 1E and 1F). Thus, GCs decrease serotonin synthesis in beta cells through decreased Tph1 and 2 expression.

### GCs inhibit prolactin or exendin-4 stimulation of Tph1 and Tph2 in beta cells

**Fig 2:**
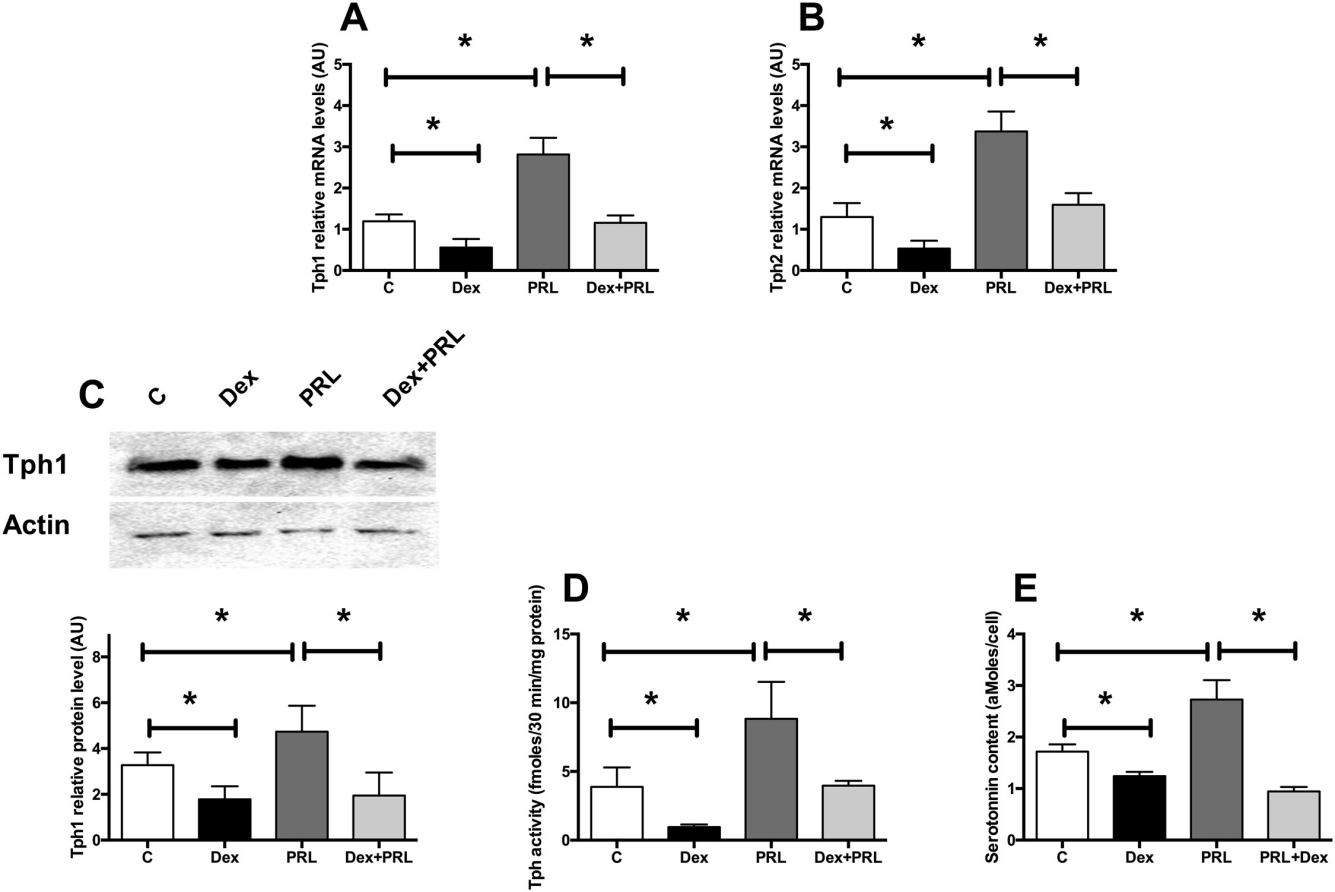
GCs inhibit prolactin activation of Tph1 and Tph 2 in beta cells. (A) Tph1 and (B) Tph 2 expression in MIN6 cells cultured in control conditions (white bars) or treated with Dexamethasone 10^−7^ M (black bars), with prolactin (100 ng/ml) (dark grey bars) or with both for 24h (light grey bars). (C) Representative immunoblot for Tph1 and Actin on protein extracts from MIN6 cells under the same conditions (upper panel) and quantification of the signals (lower panel). (D) Tph activity in MIN6 cells in the same conditions. (E) Serotonin contents measured in MIN6 cells in the same conditions. Results are expressed as means ± SD for n = 6 independent experiments. * p<0.05 and ** p<0.01 when comparing the different groups using an ANOVA test.

Prolactin (PRL) and other lactogen hormones are known to activate Tph1 and Tph2 expression as well as serotonin synthesis in beta cells [14, 15]. We investigated whether GCs counteracted the PRL-stimulated serotonin increase. In MIN6 cells, a 24h-PRL treatment increased Tph1 and Tph2 mRNA (Fig 2A and 2B, respectively) and Tph1 protein level (Fig 2C) as expected. Interestingly, Dex treatment prevented the PRL-induced Tph1 and Tph2 increase both at the mRNA (Fig 2A and 2B) and also protein level for Tph1 (Fig 2C). These modifications led to similar changes of Tph activity (Fig 2D) and serotonin contents (Fig 2E). Thus, GCs prevent the stimulatory effect of PRL on serotonin synthesis.

**Fig 3:**
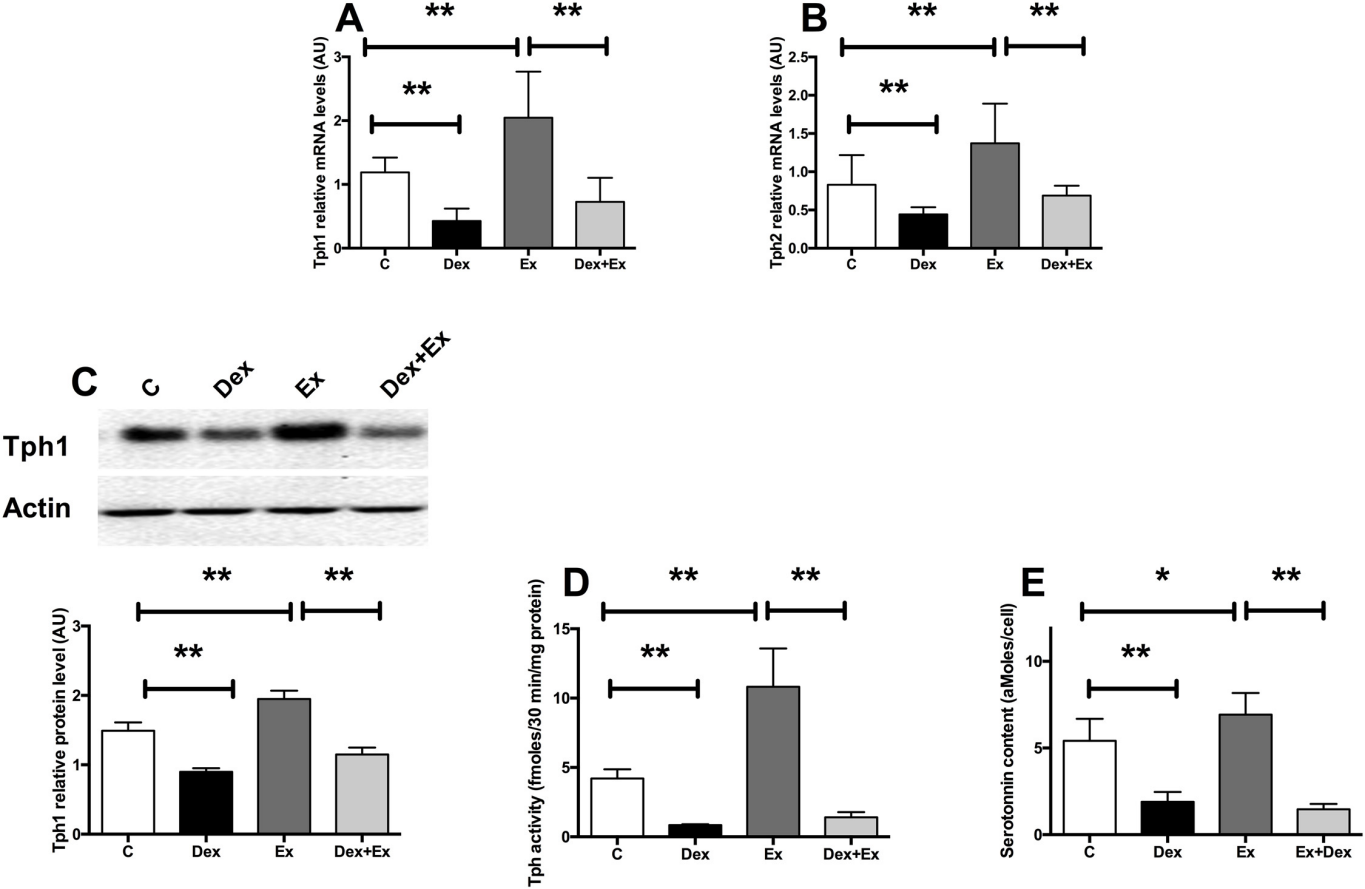
GCs inhibit exendin-4 activation of Tph1 and Tph 2 in beta cells. (A) Tph1 and (B) Tph2 mRNA levels in MIN6 cells cultured in control conditions (white bars) or treated with 10^−7^M Dexamethasone (black bars), with 100 ng/ml exendin-4 (Ex) (dark grey bars) or with both for 24h (light grey bars). (C) Representative immunoblot for Tph1 and Actin on protein extracts from MIN6 cells under the same conditions (upper panel) and quantification of the signals (lower panel). (D) Tph activity in MIN6 cells in the same conditions. (E) Serotonin contents measured in the same conditions. Results are expressed as means ± SD for n = 6 independent experiments. * p<0.05 ** p<0.01 and *** p<0.001 when comparing the different groups using a ANOVA test.

As PRL, Glucagon-like Peptide 1 (GLP-1) is one of the hormones able to stimulate beta-cell growth. We thus asked whether GLP-1 could also stimulate serotonin synthesis in beta cells. In MIN6 cells, a 24h treatment with the GLP-1 analog exendin-4 (Ex4) increased Tph1 and Tph2 mRNA levels (Fig 3A and 3B, respectively), Tph1 protein level (Fig 3C), Tph activity (Fig 3D), resulting in increased serotonin content (Fig 3E). Because GCs can counteract the effect of PRL on serotonin, we asked whether it would also block the effects of GLP-1. Similarly to what we observed with PRL induction, Dex treatment prevented Ex4-induced increase in Tph1 and Tph2 mRNA levels (Fig 3A and 3B respectively), Tph1 protein level (Fig 3C), Tph activity (Fig 3D) and serotonin content (Fig 3E). We thus showed a previously unknown stimulation of serotonin synthesis by Ex4 suggesting that GLP-1 effect on beta cells might be mediated through serotonin up-regulation. Also, similarly to PRL, Ex4 effects on serotonin were blocked by GCs.

### In vivo GLP-1 administration leads to a stimulation of serotonin synthesis in islets which is inhibited by GCs

**Fig 4:**
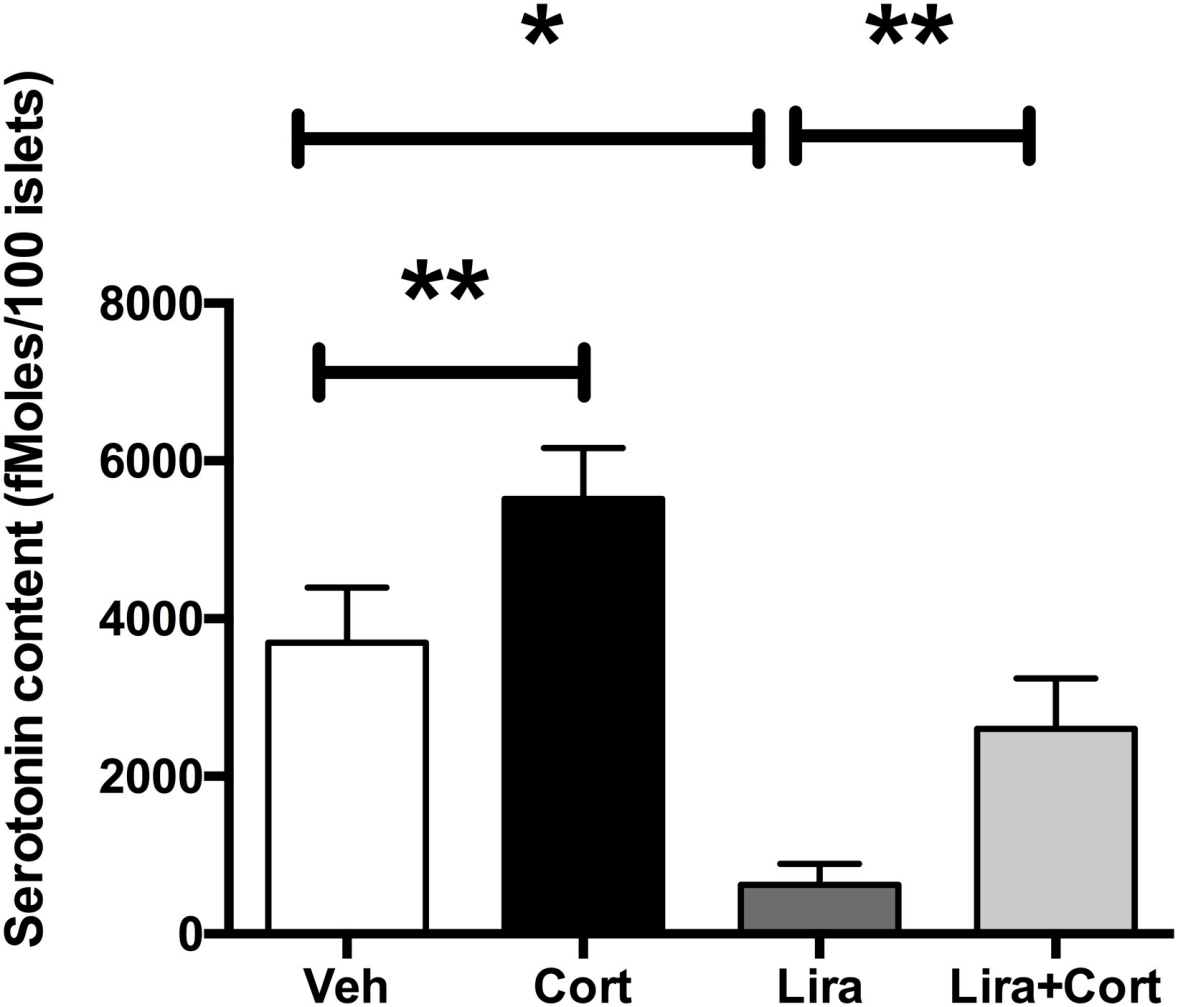
GCs inhibit liraglutide increases of serotonin contents in vivo. Serotonin contents measured on isolated islets of mice treated with vehicle only (VEH, white bar), corticosterone (Cort, black bar), liraglutide (Lira, dark grey) or both corticosterone and liraglutide (Lira+Cort, light gray) for 4 weeks. Results are expressed as means ± SD for n = 5 mice in each group. * p<0.05 ** and p<0.01 when comparing the different groups using a ANOVA test.

To further explore how GCs and GLP-1 regulate serotonin synthesis in beta cells, we treated mice for 4 weeks with corticosterone or with a GLP-1 analog liraglutide or with both molecules. At the end of the treatment, islets were isolated and serotonin contents were assayed. We observed that in vivo GCs treatment decreased serotonin contents in islets (Fig 4). When mice were treated with liraglutide only, increased serotonin contents were observed. Finally, GCs treatment prevented the liraglutide-induced increase of serotonin contents. Therefore, serotonin contents in islets in vivo are also under control of GCs and GLP-1R activation, as demonstrated in vitro.

### Increased Tph1 and Tph2 expression and serotonin synthesis in islets from GR^-^ mice

**Fig 5:**
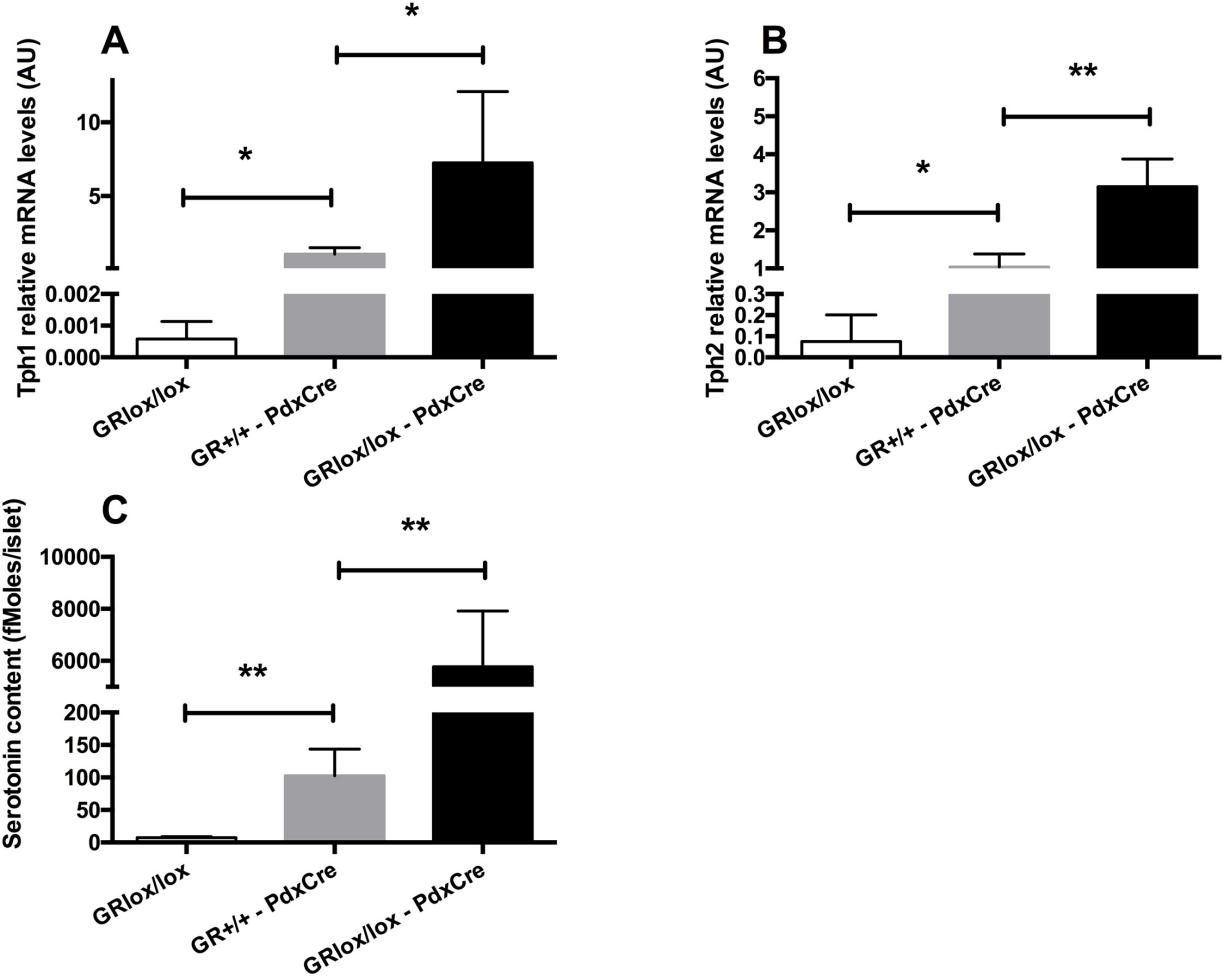
The deletion of the GR in the pancreas increases serotonin synthesis is islets. (A) Tph1, (B) Tph2 mRNA levels and (C) serotonin contents in islets from control mice (GR^Lox/Lox^, white bars), mice that carry the PdxCre transgene (GR^+/+^—PdxCre, gray bars) and mice that are deleted for the GR in the pancreas (GR^Lox/Lox^–PdxCre, black bars). Values are means ± SD. * p<0.05, ** p<0.01 when comparing GR^-^ to control mice using a non parametric Mann-Whitney test (n = 3–4 per group).

To confirm the regulation of serotonin synthesis by GCs, we measured serotonin contents and Tph expression in islets from mice that are mutated for the GR in the pancreas. These mice were generated using mice that express the Cre recombinase under the control of the Pdx1 promoter [26] crossed with GR loxed alleles [27]. A recent article reported that several mice that express the Cre in the pancreas have increased serotonin contents in islets because of the presence of a hGH minigene [21], including the Pdx1-Cre used in our study. Therefore, we chose to measure serotonin in control mice (GR^Lox/Lox^), in mice carrying the PdxCre transgene in the absence of the loxed GR alleles (GR^+/+^—PdxCre) and in mice having floxed GR alleles and carrying the PdxCre transgene (GR^Lox/Lox^–PdxCre). mRNA levels of Tph1 (Fig 5A) and Tph2 (Fig 5B) and serotonin contents (Fig 5C) were clearly increased in islets from GR^+/+^—PdxCre in comparison to control GR^Lox/Lox^ mice, confirming that the PdxCre transgene stimulates serotonin synthesis by itself. Yet, Tph1 and Tph2 mRNA levels were further increased in mice deleted for the GR in the pancreas (GR^Lox/Lox^–PdxCre) when compared to mice carrying solely the PdxCre transgene, leading to a huge increase of serotonin content. Thus, this Cre/hGH transgene led to an increase of serotonin content in islets but the GR deletion led to further increase of serotonin in islets, demonstrating that GCs really control serotonin synthesis in beta cells.

## Discussion

GCs are key regulators of glucose metabolism. They also play specific roles on beta cells since they induce beta-cell apoptosis, decrease beta-cell mass and inhibit insulin secretion, but the molecular mechanisms are still unclear [4]. We provide here original evidence that relate GCs and serotonin synthesis, a modulator of insulin secretion and beta-cell mass. Our results show that both in vitro and in vivo, GCs exposure inhibits Tph1 and 2 expression resulting in a decrease of serotonin synthesis in islets. We provide further evidence that Tph1 and Tph2 expression is under the control of GCs in beta cells since their expression is increased in islets from GR^-^ mice leading to a dramatic augmentation of serotonin content. Furthermore, beta cells exposed in vitro to GCs display also decreased Tph1 and Tph2 expression with reduced serotonin synthesis and content. Finally, GCs also blocked the stimulation of serotonin synthesis by PRL and the GLP-1 analog exendin-4. These results definitively demonstrate that serotonin synthesis is a major target of GCs in beta cells.

Recent data showed that serotonin is a major regulator of both beta-cell mass and function [14, 15]. Serotonin synthesis in beta cells is under the positive control of lactogen hormones [14]. Here, we demonstrate that other hormones, namely GCs, also control albeit negatively serotonin synthesis in beta cells. Such serotonin inhibition by GCs has been previously described in the brain: indeed, in the raphe nuclei GCs inhibit Tph2 expression, the predominant central nervous system (CNS) Tph isoform. This result suggested that elevated GCs may play a role in psychiatric diseases involving serotonin, such as depression. Clinical and preclinical studies have gathered evidence that stress response alterations play a major role in the development of major depression [28, 29]. Decrease in serotonin circulating levels has been also documented in this condition[30]. Since people suffering from depression have a higher risk to develop T2D [31], inhibition of serotonin by GCs both in neurons and beta cells might explain the association between diabetes and depression.

Interestingly, in the zebra fish whole embryo, the knockdown of GR using morpholino was associated with up-regulated Tph 2 expression [18] in line with our observation in GR^-^ islets. Thus, the control of serotonin synthesis by GCs may not be restricted to beta cells but may also extend to other tissues that produce serotonin. The conservation of Tph1 and 2 inhibitions by GCs between organs and species highlights the importance of this regulation. Further investigations are required to decipher how GCs regulate Tph1 and 2 expression. Since the GR is a transcription factor that binds to specific DNA sequences, the use of chromatin immunoprecipitation (ChIP) with a specific anti-GR antibody would allow for the identification of GCs response elements in the promoters of Tph1 and 2. Moreover, experiments performed with cloned promoters in reporter plasmid and deletion mutants would help identify the exact sequences that are crucial for GCs effects on Tph1 and 2 promoters.

Our results raise the question of the physiological and pathophysiological roles of serotonin regulation by ss. This question is particularly relevant since recent data suggested that serotonin is actually a major regulator of both beta-cell mass and function. First, a study showed that during pregnancy, lactogenic hormones induce Tph1 expression and serotonin synthesis in islets [14]. Serotonin, in turn, stimulates beta-cell proliferation through a paracrine-autocrine activation of the serotonin receptor 2b. However, a more recent study has shown that mice lacking Tph1 and unable to produce serotonin in beta cells or other peripheral tissues present normal beta cells proliferation during pregnancy, thus questioning the exact role of serotonin increase during pregnancy [32]. So far, the effects of the down-regulation of serotonin synthesis by GCs on beta-cell mass remain to be documented. One could speculate that there is a causal relationship between this down regulation and the decrease of beta cells mass due to GCs administration [33]. In addition, serotonin signaling may also directly affect insulin secretion independently of its effect on beta cell mass. A recent study demonstrated that serotonin regulates insulin secretion through a dual mode: intracellular activation through serotonylation, which consist on the addition of a serotonin moiety as a post traductional modification of key proteins involved in insulin granules fusion in beta cells, and extracellular inhibition through binding to the serotonin receptor 1a [15]. Insulin secretion inhibition by GCs may also be due to the decrease of serotonin synthesis in islet.

We show here that GCs decrease serotonin contents in vitro in beta cells and isolated islets. This decrease may impair insulin secretion since serotonin is crucial for proper insulin secretion. Yet, in contrast to in vitro studies, experiments with GCs treatment in vivo displayed different results: GCs treatment results in increase insulin secretion capacity and increased beta-cell mass [34]. We think that such discrepancy originates from the fact that GCs act directly on beta cells in vitro while in vivo, GCs act on beta cells and also on other tissues, such as muscle, liver and adipose tissue, where they induce insulin resistance. Therefore, results on beta cells obtained after GCs treatment in vivo mostly reflect beta-cell adaptation that is secondary to insulin resistance. Interestingly, in the present study, we observed decreased serotonin contents after GCs treatment in vitro and in vivo, suggesting that this regulation is a direct effect of GCs.

Mice deleted for the GR in beta cells using the PdxCre transgene present with increased beta-cell mass [33]. Here we showed that these mice also show increased expression of both Tph1 and 2 leading to increased serotonin contents in islets. Such serotonin increase may be involved in this beta-cell mass expansion similarly to what has been described during pregnancy in mice [14]. Interestingly, these mice also present mild glucose intolerance with a reduced insulin secretion (data not shown). Whether this phenotype is to be linked with the increased serotonin also remains to be elucidated.

Finally, we confirmed that prolactin stimulates serotonin synthesis in beta cells. Also, we demonstrated that the GLP-1 analog exendin-4 stimulates the expression of Tph1 and Tph2 and serotonin synthesis in beta cells, a result still unheard of. In vivo, liraglutide treatment led to increased serotonin contents in islets. Since GLP-1 is known to increase beta- cell mass in rodents [35] and also, more recently, in humans [36], our result suggests that serotonin could be a mediator of this effect. Moreover, since GLP-1 is known to increase insulin secretion in response to glucose, our results suggest that such effect may be mediated through serotonin augmentation. Interestingly, we found that the GLP-1 stimulatory signal on serotonin synthesis is blocked by the GCs. These results suggest that patients with type 2 diabetes and treated with GLP-1 analogs should be cautious about undergoing GCs treatment that may block beneficiary effects of GLP-1 on beta cells, as previously reported [37]. Interestingly, a similar stimulation of serotonin by GLP-1 has been identified in the brain [38], suggesting that this effect is conserved between beta cells and neurons. Moreover, the up-regulation of Tph1 and 2 by GLP-1 is likely to be mediated through cAMP production since the activation of the GLP-1 receptor by its agonists results in increased cAMP production [39] that has been shown to be involved in the GLP-1-induced increased insulin mRNA. Interestingly, cAMP response element binding activity has been described in the promoter of Tph2 in neurons of the cortex and hippocampus [40], suggesting that similar regulation takes place in beta cells.

In conclusion, our results demonstrate for the first time the capacity of GCs to down-regulate Tph1 and Tph2 expression and serotonin synthesis in beta cells. GCs counteract the stimulatory effects of PRL and GLP-1 on serotonin synthesis. We propose that this regulation participates to the deleterious effects of GCs on beta cells and may explain the insulin secretion defect observed in situations of excess GCs concentrations and more generally in type 2 diabetes.

## Supporting Information

S1 Dataset Raw data for Tph activity, serotonin content measurements, qPCR for Tph1 and Tph2 and Western blots for Tph1. (XLSX)

## References

[1] Pivonello R, De Leo M, Vitale P, Cozzolino A, Simeoli C, De Martino MC, et al Pathophysiology of diabetes mellitus in Cushing's syndrome. Neuroendocrinology. 2010;92 Suppl 1:77–81. Epub 2010/09/21. 10.1159/000314319 [pii]. . 20829623

[2] Resmini E, Minuto F, Colao A, Ferone D. Secondary diabetes associated with principal endocrinopathies: the impact of new treatment modalities. Acta Diabetol. 2009;46(2):85–95. Epub 2009/03/27. 10.1007/s00592-009-0112-9 . 19322513

[3] Di Dalmazi G, Pagotto U, Pasquali R, Vicennati V. Glucocorticoids and type 2 diabetes: from physiology to pathology. J Nutr Metab. 2012;2012:525093 Epub 2013/01/15. 10.1155/2012/525093 23316348 PMC3536319

[4] van Raalte DH, Ouwens DM, Diamant M. Novel insights into glucocorticoid-mediated diabetogenic effects: towards expansion of therapeutic options? EurJClinInvest. 2009;39(2):81–93. VANRAALTE2009. 10.1111/j.1365-2362.2008.02067.x 19200161

[5] Lambillotte C, Gilon P, Henquin JC. Direct glucocorticoid inhibition of insulin secretion. An in vitro study of dexamethasone effects in mouse islets. J Clin Invest. 1997;99(3):414–23. Epub 1997/02/01. 10.1172/JCI119175 9022074 PMC507814

[6] Weinhaus AJ, Bhagroo NV, Brelje TC, Sorenson RL. Dexamethasone counteracts the effect of prolactin on islet function: implications for islet regulation in late pregnancy. Endocrinology. 2000;141(4):1384–93. Epub 2000/04/04. 10.1210/endo.141.4.7409 . 10746642

[7] Adcock IM, Caramori G, Ito K. New insights into the molecular mechanisms of corticosteroids actions. Curr Drug Targets. 2006;7(6):649–60. Epub 2006/06/22. . 16787166 10.2174/138945006777435344

[8] Gremlich S, Roduit R, Thorens B. Dexamethasone induces posttranslational degradation of GLUT2 and inhibition of insulin secretion in isolated pancreatic beta cells. Comparison with the effects of fatty acids. J Biol Chem. 1997;272(6):3216–22. Epub 1997/02/07. . 9013557 10.1074/jbc.272.6.3216

[9] Ohneda M, Johnson JH, Inman LR, Unger RH. GLUT-2 function in glucose-unresponsive beta cells of dexamethasone-induced diabetes in rats. J Clin Invest. 1993;92(4):1950–6. Epub 1993/10/01. 10.1172/JCI116788 8408647 PMC288361

[10] Borboni P, Porzio O, Magnaterra R, Fusco A, Sesti G, Lauro R, et al Quantitative analysis of pancreatic glucokinase gene expression in cultured beta cells by competitive polymerase chain reaction. Mol Cell Endocrinol. 1996;117(2):175–81. Epub 1996/03/25. 0303720795037454 [pii]. . 8737377 10.1016/0303-7207(95)03745-4

[11] Ullrich S, Berchtold S, Ranta F, Seebohm G, Henke G, Lupescu A, et al Serum- and glucocorticoid-inducible kinase 1 (SGK1) mediates glucocorticoid-induced inhibition of insulin secretion. Diabetes. 2005;54(4):1090–9. Epub 2005/03/29. 54/4/1090 [pii]. . 15793248 10.2337/diabetes.54.4.1090

[12] Hamamdzic D, Duzic E, Sherlock JD, Lanier SM. Regulation of alpha 2-adrenergic receptor expression and signaling in pancreatic beta-cells. Am J Physiol. 1995;269(1 Pt 1):E162–71. Epub 1995/07/01. . 7631772 10.1152/ajpendo.1995.269.1.E162

[13] Zawalich WS, Tesz GJ, Yamazaki H, Zawalich KC, Philbrick W. Dexamethasone suppresses phospholipase C activation and insulin secretion from isolated rat islets. Metabolism. 2006;55(1):35–42. Epub 2005/12/06. S0026-0495(05)00273-8 [pii] 10.1016/j.metabol.2005.06.023 . 16324917

[14] Kim H, Toyofuku Y, Lynn FC, Chak E, Uchida T, Mizukami H, et al Serotonin regulates pancreatic beta cell mass during pregnancy. Nat Med. 2010;16(7):804–8. Epub 2010/06/29. nm.2173 [pii] 10.1038/nm.2173 20581837 PMC2921604

[15] Paulmann N, Grohmann M, Voigt JP, Bert B, Vowinckel J, Bader M, et al Intracellular serotonin modulates insulin secretion from pancreatic beta-cells by protein serotonylation. PLoS Biol. 2009;7(10):e1000229 Epub 2009/10/28. 10.1371/journal.pbio.1000229 19859528 PMC2760755

[16] El-Merahbi R, Loffler M, Mayer A, Sumara G. The roles of peripheral serotonin in metabolic homeostasis. FEBS Lett. 2015;589(15):1728–34. Epub 2015/06/14. 10.1016/j.febslet.2015.05.054 S0014-5793(15)00455-X [pii]. . 26070423

[17] Jaim-Etcheverry G, Zieher LM. Electron microscopic cytochemistry of 5-hydroxytryptamine (5-HT) in the beta cells of guinea pig endocrine pancreas. Endocrinology. 1968;83(5):917–23. Epub 1968/11/01. 10.1210/endo-83-5-917 . 4879454

[18] Nesan D, Vijayan MM. The transcriptomics of glucocorticoid receptor signaling in developing zebrafish. PLoS One. 2013;8(11):e80726 Epub 2013/12/19. 10.1371/journal.pone.0080726 PONE-D-13-22836 [pii]. 24348914 PMC3858477

[19] Miyazaki J, Araki K, Yamato E, Ikegami H, Asano T, Shibasaki Y, et al Establishment of a pancreatic beta cell line that retains glucose-inducible insulin secretion: special reference to expression of glucose transporter isoforms. Endocrinology. 1990;127(1):126–32. MIYAZAKI1990A. 2163307 10.1210/endo-127-1-126

[20] Valtat B, Dupuis C, Zenaty D, Singh-Estivalet A, Tronche F, Breant B, et al Genetic evidence of the programming of beta cell mass and function by glucocorticoids in mice. Diabetologia. 2011;54(2):350–9. VALTAT2011. 10.1007/s00125-010-1898-2 20857084

[21] Brouwers B, de Faudeur G, Osipovich AB, Goyvaerts L, Lemaire K, Boesmans L, et al Impaired Islet Function in Commonly Used Transgenic Mouse Lines due to Human Growth Hormone Minigene Expression. Cell Metab. 2014;20(6):979–90. Epub 2014/12/04. 10.1016/j.cmet.2014.11.004 S1550-4131(14)00501-4 [pii]. . 25470546 PMC5674787

[22] Fransson L, Dos Santos C, Wolbert P, Sjoholm A, Rafacho A, Ortsater H. Liraglutide counteracts obesity and glucose intolerance in a mouse model of glucocorticoid-induced metabolic syndrome. Diabetol Metab Syndr. 2014;6(1):3 Epub 2014/01/16. 10.1186/1758-5996-6-3 [pii]. 24423471 PMC3905931

[23] Valtat B, Riveline JP, Zhang P, Singh-Estivalet A, Armanet M, Venteclef N, et al Fetal PGC-1alpha overexpression programs adult pancreatic beta-cell dysfunction. Diabetes. 2013;62(4):1206–16. Epub 2013/01/01. 10.2337/db12-0314 [pii]. 23274887 PMC3609553

[24] Kema IP, Schellings AM, Hoppenbrouwers CJ, Rutgers HM, de Vries EG, Muskiet FA. High performance liquid chromatographic profiling of tryptophan and related indoles in body fluids and tissues of carcinoid patients. Clin Chim Acta. 1993;221(1–2):143–58. Epub 1993/11/30. . 7512001 10.1016/0009-8981(93)90029-4

[25] Khan IA, Thomas P. Disruption of neuroendocrine control of luteinizing hormone secretion by aroclor 1254 involves inhibition of hypothalamic tryptophan hydroxylase activity. Biol Reprod. 2001;64(3):955–64. Epub 2001/02/24. . 11207213 10.1095/biolreprod64.3.955

[26] Herrera PL. Adult insulin- and glucagon-producing cells differentiate from two independent cell lineages. Development. 2000;127(11):2317–22. Epub 2000/05/11. . 10804174 10.1242/dev.127.11.2317

[27] Tronche F, Kellendonk C, Reichardt HM, Schutz G. Genetic dissection of glucocorticoid receptor function in mice. Curr Opin Genet Dev. 1998;8(5):532–8. Epub 1998/10/31. S0959-437X(98)80007-5 [pii]. . 9794823 10.1016/s0959-437x(98)80007-5

[28] Clark JA, Flick RB, Pai LY, Szalayova I, Key S, Conley RK, et al Glucocorticoid modulation of tryptophan hydroxylase-2 protein in raphe nuclei and 5-hydroxytryptophan concentrations in frontal cortex of C57/Bl6 mice. Mol Psychiatry. 2008;13(5):498–506. Epub 2007/07/12. 4002041 [pii] 10.1038/sj.mp.4002041 17622221 PMC3392182

[29] Pariante CM, Lightman SL. The HPA axis in major depression: classical theories and new developments. Trends Neurosci. 2008;31(9):464–8. Epub 2008/08/05. 10.1016/j.tins.2008.06.006 S0166-2236(08)00164-1 [pii]. . 18675469

[30] Hirschfeld RM. History and evolution of the monoamine hypothesis of depression. J Clin Psychiatry. 2000;61 Suppl 6:4–6. Epub 2000/04/25. . 10775017

[31] Cappuccio FP, D'Elia L, Strazzullo P, Miller MA. Quantity and quality of sleep and incidence of type 2 diabetes: a systematic review and meta-analysis. Diabetes Care. 2010;33(2):414–20. Epub 2009/11/17. 10.2337/dc09-1124 dc09-1124 [pii]. 19910503 PMC2809295

[32] Schraenen A, Lemaire K, de Faudeur G, Hendrickx N, Granvik M, Van Lommel L, et al Placental lactogens induce serotonin biosynthesis in a subset of mouse beta cells during pregnancy. Diabetologia. 2010;53(12):2589–99. Epub 2010/10/13. 10.1007/s00125-010-1913-7 20938637 PMC2974930

[33] Gesina E, Tronche F, Herrera P, Duchene B, Tales W, Czernichow P, et al Dissecting the role of glucocorticoids on pancreas development. Diabetes. 2004;53(9):2322–9. GESINA2004. 15331541 10.2337/diabetes.53.9.2322

[34] Protzek AO, Costa-Junior JM, Rezende LF, Santos GJ, Araujo TG, Vettorazzi JF, et al Augmented beta-Cell Function and Mass in Glucocorticoid-Treated Rodents Are Associated with Increased Islet Ir-beta /AKT/mTOR and Decreased AMPK/ACC and AS160 Signaling. Int J Endocrinol. 2014;2014:983453 Epub 2014/10/15. 10.1155/2014/983453 25313308 PMC4182854

[35] Xu G, Stoffers DA, Habener JF, Bonner-Weir S. Exendin-4 stimulates both beta-cell replication and neogenesis, resulting in increased beta-cell mass and improved glucose tolerance in diabetic rats. Diabetes. 1999;48(12):2270–6. Epub 1999/12/02. . 10580413 10.2337/diabetes.48.12.2270

[36] Butler AE, Campbell-Thompson M, Gurlo T, Dawson DW, Atkinson M, Butler PC. Marked Expansion of Exocrine and Endocrine Pancreas with Incretin Therapy in Humans with increased Exocrine Pancreas Dysplasia and the potential for Glucagon-producing Neuroendocrine Tumors. Diabetes. 2013 Epub 2013/03/26. db12-1686 [pii] 10.2337/db12-1686 . 23524641 PMC3712065

[37] van Raalte DH, van Genugten RE, Linssen MM, Ouwens DM, Diamant M. Glucagon-like peptide-1 receptor agonist treatment prevents glucocorticoid-induced glucose intolerance and islet-cell dysfunction in humans. Diabetes Care. 2011;34(2):412–7. Epub 2011/01/11. dc10-1677 [pii] 10.2337/dc10-1677 21216851 PMC3024359

[38] Brunetti L, Orlando G, Recinella L, Leone S, Ferrante C, Chiavaroli A, et al Glucagon-like peptide 1 (7–36) amide (GLP-1) and exendin-4 stimulate serotonin release in rat hypothalamus. Peptides. 2008;29(8):1377–81. Epub 2008/05/27. 10.1016/j.peptides.2008.04.007 S0196-9781(08)00180-0 [pii]. . 18502539

[39] Drucker DJ, Philippe J, Mojsov S, Chick WL, Habener JF. Glucagon-like peptide I stimulates insulin gene expression and increases cyclic AMP levels in a rat islet cell line. Proc Natl Acad Sci U S A. 1987;84(10):3434–8. Epub 1987/05/01. 3033647 10.1073/pnas.84.10.3434 PMC304885

[40] Garcia-Osta A, Del Rio J, Frechilla D. Increased CRE-binding activity and tryptophan hydroxylase mRNA expression induced by 3,4-methylenedioxymethamphetamine (MDMA, "ecstasy") in the rat frontal cortex but not in the hippocampus. Brain Res Mol Brain Res. 2004;126(2):181–7. Epub 2004/07/14. 10.1016/j.molbrainres.2004.04.006 S0169328X04001950 [pii]. . 15249142

