# Correction: Glucocorticoids Inhibit Basal and Hormone-Induced Serotonin Synthesis in Pancreatic Beta Cells

**DOI:** 10.1371/journal.pone.0155174

**Published:** 2016-05-04

**Authors:** Moina Hasni Ebou, Amrit Singh-Estivalet, Jean-Marie Launay, Jacques Callebert, François Tronche, Pascal Ferré, Jean-François Gautier, Ghislaine Guillemain, Bernadette Bréant, Bertrand Blondeau, Jean-Pierre Riveline

Fig 4 and its caption appear incorrectly in the published article. Please see the correct Fig 4 and its caption here.

**Fig 4:**
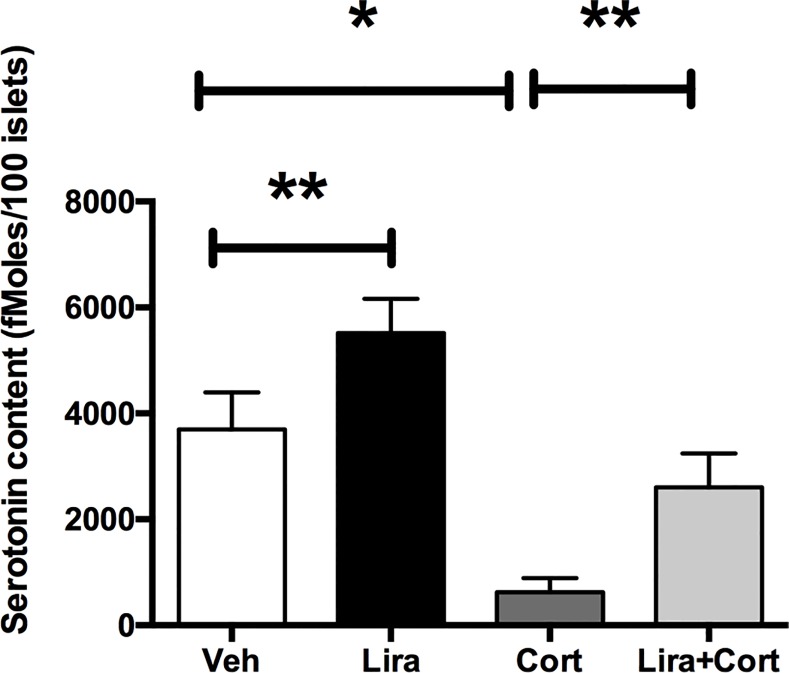
GCs inhibit liraglutide-induced increase of serotonin contents in vivo. Serotonin contents measured on isolated islets of mice treated with vehicle only (Veh, white bar), liraglutide (Lira, black bar), corticosterone (Cort, dark grey) or both liraglutide and corticosterone (Lira+Cort, light gray) for 4 weeks. Results are expressed as means ± SD for n = 5 mice in each group. * p<0.05 ** and p<0.01 when comparing the different groups using a ANOVA test.
